# “We Were Still Left in the Back Field, Not Knowing”: Pediatric Cancer Patients and Parents Describe Obstacles to Prognostic Communication

**DOI:** 10.1002/cam4.70810

**Published:** 2025-04-08

**Authors:** Adriana Areizaga Ayala, Harmony Farner, Shoshana Mehler, Caroline Christianson, Tara M. Brinkman, Justin N. Baker, Pamela S. Hinds, Jennifer W. Mack, Erica C. Kaye

**Affiliations:** ^1^ School of Medicine Universidad Central del Caribe Bayamón Puerto Rico; ^2^ Department of Oncology St Jude Children's Research Hospital Memphis Tennessee USA; ^3^ Division of Pediatric Hematology/Oncology NYU Langone Health New York City New York USA; ^4^ Department of Psychology and Biobehavioral Sciences St Jude Children's Research Hospital Memphis Tennessee USA; ^5^ Department of Epidemiology and Cancer Control St Jude Children's Research Hospital Memphis Tennessee USA; ^6^ Department of Pediatrics Stanford University Palo Alto California USA; ^7^ Department of Nursing Science, Professional Practice & Quality Children's National Hospital Washington DC USA; ^8^ Department of Pediatrics School of Medicine and Health Sciences The George Washington University Washington DC USA; ^9^ Department of Pediatric Oncology Dana‐Farber Cancer Institute Boston Massachusetts USA; ^10^ Division of Population Sciences' Center for Outcomes and Policy Research Dana‐Farber Cancer Institute Boston Massachusetts USA

**Keywords:** adolescents and young adults, cancer, children, obstacles, parents, prognostic communication

## Abstract

**Purpose:**

Patient/parent perceptions of poor‐quality prognostic disclosure have not been well described, and these data offer important lessons to shape clinical practice and communication skills training. In this study, we aimed to characterize patient/parent negative experiences with prognostic communication to inform future efforts to improve how clinicians disclose prognosis.

**Patients and Methods:**

Semistructured interviews were conducted with a purposeful sample of pediatric cancer patients (*n* = 25) and parents (*n* = 40) across different timepoints in the progressive illness course extending into bereavement. Interviews were audio‐recorded, transcribed, and de‐identified for rapid qualitative analysis, in which multiple analysts used a standardized episode summary template to extract raw data specific to patient/parent narratives about prognostic disclosure experiences. Analysts engaged independently and collaboratively as a team in reflexive memo writing to identify negative experiences with prognostic communication, followed by team discussion to generate concepts and synthesize those concepts into themes.

**Results:**

More than half of participants (59%) described negative experiences with prognostic disclosure, with parents highlighting distressing communication experiences more often than patients (parents: 32/40, 80% vs. patients: 6/25, 24%). Across patient/parent narratives, three main themes underpinned the perception of poor‐quality prognostic communication: (1) insufficient information, (2) overwhelming or contradictory information, and (3) absence of person‐centered connection.

**Conclusion:**

Many patients/parents perceived prognostic disclosure to be suboptimal and identified specific features underpinning poor‐quality prognostic communication. These findings will inform future collaborative research with patients, parents, and multidisciplinary clinicians to codesign an intervention that individualizes prognostication to align with patient/parent preferences for receiving information and fostering connection.

## Graphical abstract

Patients with cancer and their families have important lessons to teach oncology clinicians about how to improve care. In this study, we asked pediatric patients and parents to reflect on how their doctors talked about prognosis to help us identify ways to improve this process. We learned that when patients and parents feel that the doctor did not discuss prognosis in a good way, it was usually due to (1) insufficient information, (2) overwhelming or contradictory information, and (3) absence of person‐centered connection. These findings will help guide communication training and future research to improve communication.

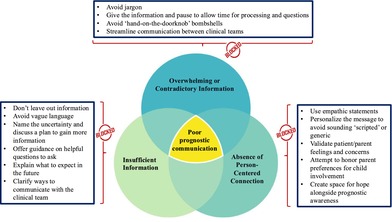

## 1 Introduction

Compassionate, timely, person‐centered communication, in which clinicians recognize and respond to the individualized needs, values, and preferences of patients and caregivers, is a critical component of high‐quality pediatric cancer care [1]. In the setting of advanced childhood cancer, effective prognostic communication, or discussion about how a disease may impact one's future life, can help patients, parents, and oncologists align their understanding of the likelihood of cure [2]. Moreover, high‐quality prognostic communication can facilitate conversations about goals of care [3], support parents in transitioning from curative to comfort‐focused treatment [4], create opportunities for reframing hope [5], and mitigate complicated bereavement [6].

A growing body of data suggests that most pediatric patients with cancer and their parents want clear, compassionate, and frequent information about prognosis [7, 8]. Unfortunately, gaps persist in the provision of transparent, timely prognostic disclosure [9], with oncologists often softening or deferring clear information about the likelihood of cure or implications for the future [2, 10, 11]. Clinicians face barriers to the provision of high‐quality prognostic communication across individual, team, organizational, community, and policy levels [12], with the advent of novel therapeutics further complicating prognostication [13, 14].

In response to these challenges, the National Cancer Institute (NCI) identified patient‐centered communication as a priority to promote healing and reduce suffering, endorsing six core functions of communication to guide bedside care and provide infrastructure for scientific inquiry, including exchanging information, responding to emotions, managing uncertainty, fostering healing relationships, making decisions, and enabling patient self‐management [15].

Clinicians and researchers in pediatrics further broadened this definition to include the patient–caregiver–clinician triad, emphasizing the value of a person‐(rather than only patient‐) centered approach [16, 17].Within the last decade, systematic reviews have described the expansion of communication science in pediatric oncology [18, 19, 20], and broad functions of communication in childhood cancer care have been described, largely affirming the NCI core domains and establishing infrastructure for best practices in pediatric cancer communication [21, 22].

While several studies have described patient/parent perceptions of the gold standard for general communication [23, 24], fewer studies have characterized quality communication specifically in the context of prognostic disclosure. These studies offer guidance from pediatric patients and parents on “best” practices for prognostic communication, including setting the scene, personalizing language, empowering the patient/family, providing anticipatory guidance, leaving room for hope, and providing information early and often [25, 26].

Notably, little research to date has explored patient/parent perspectives on what *not* to do or say when navigating difficult prognostic conversations. Avoiding suboptimal approaches requires more than the inverse of best practices—missteps in prognostic disclosure offer critical insights that can refine bedside communication and enhance training. To address this knowledge gap, we gathered narratives from pediatric cancer patients and parents at a center specializing in early‐phase clinical trials for relapsed/refractory disease. In this targeted analysis, we aimed to identify communication strategies perceived as ineffective or harmful by patients and parents, providing guide rails for clinicians and educators navigating challenging prognostic discussions.

## 2 Methods

The RIGHTime (Revealing Information Genuinely and Honestly Across Time) study is a longitudinal, prospective, mixed methods investigation of the “right” way to talk about prognosis in the setting of advanced childhood cancer. In the first phase of this study, interviews were conducted with patients, parents, and oncologists to gather perspectives and recommendations about person‐centered prognostic communication. The study protocol was approved by the Institutional Review Board at St. Jude Children's Research Hospital. Study methods and findings are reported in alignment with the Consolidated Criteria for Reporting Qualitative Research checklist (Appendix A) [27].

### 2.1 Eligibility, Screening, Recruitment, and Enrollment Processes

Semistructured interviews were conducted with patients and parents of children with poor prognosis cancer. Eligible patients were aged 12–25 years diagnosed with cancer with ≤ 50% odds of survival as defined by a pediatric oncologist's review of the medical record, and spoke English; patients were purposefully recruited from three different timepoints across the progressive illness trajectory: ≤ 3 months from diagnosis, ≤ 3 months from relapse/progression, and ≤ 3 months from Phase I/II trial enrollment. The 3‐month window was used to recruit relatively similar cohorts with respect to timing from key milestones, enable flexibility for the timing of informed consent, and minimize the potential for recall bias [28] (e.g., avoid some participants in a given cohort reflecting back on conversations from many months ago, while others recalled more recent conversations that were fresher in their minds).

Eligible parents spoke English and were primary caregivers of children of any age with poor prognosis cancer, and they were recruited at the same three timepoints plus an additional timepoint to include bereaved parents 6–24 months after the death of a child. The research team did not purposefully target enrollment of dyads; however, patients and parents within the same family unit were permitted to participate in the study if both wished to enroll.

Eligible participants were identified through review of patient rosters, protocol lists, and an institutional bereavement database. Purposeful sampling was used to generate a diverse sample that included participants from different ages, genders, races, ethnicities, and disease types [29]. Participants were recruited and enrolled in person or via telephone.

### 2.2 Interview Guide Development, Data Collection, and Analysis

A multidisciplinary research team with expertise in pediatric oncology, palliative care, psychology, grief and bereavement care, communication science, and qualitative research (Table 1) developed developmentally appropriate, matched interview guides for patients and parents, structured by the National Cancer Institute (NCI) six core functions of cancer communication [15]. Question prompts and probes were developed specific to each core function, eliciting patient, parent, and oncologist perspectives and recommendations regarding the “right” way to achieve high‐quality communication within a given function. Language in the bereaved parent interview guide was modified to acknowledge the death of a child; otherwise, question items remained the same across guides. The guides were then shared with LegacyVoice, an institutional listserv comprised ≥ 100 bereaved parents who partner with researchers to help develop and refine study methods and materials. An electronic survey tool was used to collect anonymous feedback about language (i.e., were question items clear and easy to understand?), content (i.e., should any questions be added or deleted?), and structure (i.e., was the order of questions logical? Was the flow of the interview easy to follow?). The guides were revised to integrate all feedback prior to finalizing documents.

**TABLE 1:** Research team attributes and qualifications.

Author	Attributes and qualifications
A.A.	Female Latina medical student with training in rapid qualitative analysis.
H.F.	Female white research associate with a Master's Degree in anthropology and expertise in qualitative research methodologies.
S.M.	Female white research associate with expertise in qualitative research methodology.
N.M.	Female Indian researcher with PhD in health services research and expertise in qualitative methodologies.
T.M.B.	Female white psychologist with PhD and clinical and research expertise in pediatric psycho‐oncology supportive care, grief, and bereavement.
J.N.B.	Male white physician–scientist with a Medical Degree, clinical and research expertise related to difficult communication in oncology, and clinical training and practice in pediatric hematology–oncology and hospice and palliative medicine.
P.H.	Female white nurse–scientist with a PhD and research expertise in stakeholder‐driven participatory research, with a focus on elevating the child and parent voice.
J.M.	Female white physician–scientist with a Medical Degree, a Master's in Public Health, research expertise in communication science, clinical training in pediatric hematology–oncology and hospice and palliative medicine, and practice in pediatric hematology–oncology.
E.K.	Female white physician–scientist with a Medical Degree, a Master's in Public Health, graduate‐level training in qualitative research methodology with a focus on communication science, and clinical training and practice in pediatric hematology–oncology and hospice and palliative medicine.
All Authors	*No members of the research team had a personal or professional relationship established with study participants prior to study commencement. During the informed consent process, research team members informed participants about the team's goals/reasons for conducting this research; individual interviewers otherwise did not share personal feelings or reasons for conducting the research with participants during the study process. All study team members share a collective interest in partnering with patients, parents, and clinicians to improve person‐centered prognostic disclosure in pediatric cancer*.

A rigorous, systematic process for training research staff to conduct sensitive interviews was previously described [26], including observation and participation in mock interviews, practice with “difficult” role‐play interviews (e.g., participant appears angry, depressed, taciturn, etc.) with third‐party review and feedback, and team meetings to discuss navigating sensitive topics, how to respond to stressful questions or comments, and practicing self‐care. Trained staff (E.K., H.F., S.M., K.B., N.M.) conducted single timepoint interviews in person in the clinic or hospital setting or via telephone, at the participants' discretion. Interviews were audio‐recorded, transcribed, and de‐identified. Field notes were also taken by the interviewer and reviewed by the research team.

This paper reports findings from patient/parent responses to the open‐ended question prompt: “Has your/your child's doctor talked about prognosis with you before?” Follow‐up probes included: “How did that experience go?”, “What was good/helpful about it?”, “What parts went well?”, “What could have been better?”, “What parts didn't go well?”

A rapid analysis approach was used to inductively derive, organize, and synthesize data across rich transcripts and generate actionable themes to inform efficient movement into the next steps toward the design and implementation of clinical intervention [30]. Responses to interview questions were analyzed using an “episode summary” template structured by NCI core communication domains (H.F., S.M., E.K.). First, analysts reviewed transcripts and conducted memo writing to begin generating de novo concepts. Then, they condensed relevant data within each NCI domain into concepts that represented distinct ideas within a given passage of transcribed text. For the first five transcripts in each cohort, at least two analysts independently generated concepts, and the research team reviewed and compared summaries, demonstrating high rates (> 90%) of concordance. The remainder of transcripts was reviewed by one analyst and audited by another, with rare variances reviewed and reconciled by the research team.

Consistent concepts emerged across patient and parent cohorts at different timepoints, prompting the research team to analyze data collectively when generating themes. Within these collective cohorts, the research team began to recognize predominantly overlapping concepts within 10–15 transcripts, with no new concepts emerging by around 15–20 transcripts. As enrollment was conducted prior to analysis, the team completed the analysis of all transcripts to honor the time invested by each participant. Analysts then transposed concepts into a matrix to facilitate horizontal analysis across participants. They categorized participants' prognostic disclosure narratives as negative or positive experiences, with the a priori goal of focusing on lessons learned from those who vulnerably shared unhelpful or harmful communication encounters. Concepts identified within the negative narratives were reviewed by the research team to characterize patterns within and between patient and parent cohorts. Through team discussion, overlapping or interrelated concepts were synthesized to generate themes.

## 3 Results

Of the 96 participants approached, 74 consented (77%), of which 65 completed interviews (68%) including 25 patients and 40 parents. Ten participants were enrolled for each of the targeted timepoints, with the exception of newly diagnosed patients, in which five patients were enrolled due to the relatively low incidence of AYA patients presenting with poor prognosis disease at the time of diagnosis. About a quarter of participants comprised patient–parent dyads (*n* = 8 dyads). Participant demographic variables are reported in Table 2. Median interview duration was 25 min for patients (19–38) and 53 min for parents (19–140).

**TABLE 2:** Participant demographic characteristics.

Participants	*n* (%)
**Parents (*n* = 40)**	
Gender	
Male	8 (20)
Female	32 (80)
Race	
White	24 (60)
Black/AA	14 (35)
Asian	1 (5)
Biracial	1 (5)
Middle Eastern	0 (0)
Ethnicity	
Hispanic/Latino	5 (13)
Non‐hispanic	35 (87)
**Patients (*n* = 25)**	
Gender	
Male	14 (56)
Female	11 (44)
Race	
White	18 (72)
Black/AA	6 (24)
Asian	0 (0)
Biracial	1 (4)
Middle Eastern	0 (0)
Ethnicity	
Hispanic/Latino	3 (12)
Non‐hispanic	22 (88)
Patient age at diagnosis	
12–14 years	13 (52)
15–17 years	7 (28)
18–20 years	3 (12)
21–25 years	2 (8)

*Note:* Nonparticipants: Of the 96 participants approached, 22 declined to participate (23%) and 9 were lost to follow‐up prior to the interview (9%) due to challenging illness circumstances or the patient dying before the interview could be scheduled. No patterns of exclusion for minoritized or underrepresented groups were identified. Specifically, of those who declined to participate, 23% were Black (compared to 35% of those who opted to participate) and 13% were Hispanic (compared to 12% of those who opted to participate).

More than half of the participants (59%) described a negative experience with prognostic disclosure, with a greater percentage of parents reporting negative experiences compared to patients (parents: 32/40, 80%; patients: 6/25, 24%). Similar numbers of participants reported negative experiences across cohorts (mean/median *n* = 8, range 6–9). Patients who shared negative experiences displayed a near bell curve of ages (mean/median 16 years, range 12–24), one‐third self‐identified as Black and none as Latino/Hispanic; most (5/6) were male, and diagnoses were evenly split between solid tumors and hematologic malignancies.

Across both patient and parent narratives, two main themes were identified underpinning the perception of poor‐quality prognostic communication: insufficient information and overwhelming or contradictory information. Parents also emphasized a third theme: Absence of person‐centered connection. No noticeable differences in themes were identified between cohorts across different timepoints. The core concepts comprising each theme are presented in Tables 3, 4, 5, alongside additional supporting quotations.

**TABLE 3:** Insufficient information as a key component of poor prognostic communication.

Concepts	Supporting quotes
Inadequate explanation or deficits in information provided	*I knew that it was difficult but I didn't understand that it was rarely cured right. I didn't understand that the only way he could ever be cancer free was if this tumor is removed. I didn't understand that it's possible this tumor may never be removed…I think it would have been easier if that had just been fully put out there initially because we were already in shock anyway* (PR14). *I like to be told anything that's going on, just like hear it from their doctors, all of it right then and there… like when I found out about transplant and all this stuff that my parents had knew about for so long and it might've been easier for me if I knew already and stuff* (PT3, age 14).
Vagueness or ambiguity of language	*Beating around the bush…kind of like not wanting to say it or making a long trail up to it…I don't want that…I just wanted to know. Just give it to me straight* (PT6, age 15). *“It's been kind of vague…it's almost like they don't want to say one way or the other…so, it's kind of like, for this moment in time, here's the situation and you don't really get much of a clear picture of what's coming or whatever's going on” (PR5)*. *I think the more that can be talked about and said I think the better. I don't think it helps to kind of try to couch it in polite terms, if you know what I mean? Like it's too vague and oblique* (PR15).
Deficits in anticipatory guidance	*“If something is gonna affect my future, [then] they should tell me how it's gonna affect my future, and if something is gonna like—the surgery is gonna cause me to have to do certain things after the surgery then they should tell me what those things are gonna be. They should've talked to me about it.”* (PT7, age 14). *How it will affect [my] child's life, will he get better, or is this something that I should prepare myself for. All they telling me is that, they going to do their best, that the chemo that he got is aggressive, and hopefully, that it'll shrink the tumor small enough for them to remove it. I try to Google it and stuff, but I can't really find too much on Google because it is rare, so I feel like [they] know more, but they're not telling me* (PR13). *Just myself as a parent, I don't know what I don't know. I might not know what to ask. I might not know what's important… don't want to end up down the road with a parent saying, “How could you not have told me this? Why did you keep this from me? I needed to know. I needed to get family in to say goodbye to my child, or I needed to change my insurance plan.” I feel like it's a medical—it's part of the scope as a medical professional, that you're trying to give someone the full picture* (PR3). *I can't imagine finding out down the road that there's some part of [child's name]'s full picture that hasn't been shared with me because I didn't know to ask* (PR16).
Inconsistent availability of clinicians	*I know you can't just reach out to doctors all the time, but I don't get a lotta time with them… sometimes it's literally they're in the room maybe under five minutes, so a little longer* (P17). *[Doctor]'s not in the office that day and all of a sudden some other doctor comes in and says, “Hey, I know she had scans today and blah, blah, blah, blah, blah.” And it's kind of like it's not the news you want to hear and it's coming from someone you have no idea who they are* (PR18).

**TABLE 4:** Overwhelming or contradictory information as a key component of poor prognostic communication.

Concepts	Supporting quotes
Complex medical terminology	*If [the oncologist could] just break it down and be like, ‘This [jargon], which means this in simpler terms’…Just so I can understand ‘cause I'm not a doctor*… (PT6 age 15). *In [another hospital], scientific terms and numbers. Here, everyday language, which I really like, and I know that's not how everybody wants it. But that really helped me a ton* (PT8, age 22). *There's a person that works with our doctor who will sometimes be in the cafeteria and he sometimes just says things in a more, I don't want to say dumb down level because it's really…it's just more to it I guess. And we'll go, oh, okay. Like that's what we were missing from the first conversation [with the oncologist]* (P5).
Excessive or incongruous content	*You know it's cancer, but what exactly it does to your body was hard to understand for a bit. I feel like no matter how they explained it to me, it seemed like it didn't make sense ‘cause it was all too much at once to take in* (PT9, age 16). *You know, it was just overwhelming to me, and my doctor [is] in the blunt category, she says, “Well, if you think that list is bad, just wait until you see the one for chemotherapy.” And I was like, “That is a patently unhelpful helpful comment right now”* (PR15). *We already had so much on our plate that we were just not ready. That's what I'm sayin’. If they would've told me she had a week to live right after him [patient's father] dyin’ a month earlier, I would have not made it through. You just have to play it by ear I think with your patient, that kinda thing ‘cause like real life happens…* (PR19).
Disjointed communication between multiple clinicians	*Sometimes you'll hear something from one doctor and then you hear the same information from another doctor said in a different way and you're like, okay, that gave me more of an idea of what's actually happening, right?* (PR5) *We do feel like there is a little bit of dysfunction in the communication that we're receiving on a day‐to‐day basis* (PR4).

**TABLE 5:** Absence of person‐centered connection as a key component of poor prognostic communication.

Concepts	Supporting quotes
Lack of empathy or impersonal disclosure	*Well, it was the way he told us. It wasn't like, ‘You have cancer. I think you should go to [hospital name]’…It was like, ‘You need to go to the hospital now…you need to go.’ He was a lot more urgent…he made it seem like we had to go right away* (PT7, age 14). *Our doctor who gave us the original diagnosis, it felt to me like she had her canned response that this is how I tell people that their child has cancer. And she hits certain points that she needs to tell you, and I didn't even get a chance to say, like, I'm not interested in numbers, you know, don't give me the numbers…* (PR10). *Health care professionals can become cold and indifferent and not realize the added trauma that you're putting on the person* (PR20).
Failure to center the parent's voice and/or preferences	*This is her fourth time with cancer…I just felt like if you would have listened to me, just listen. That's all, just listen…* (PR11). *…they dismissed her cancer symptoms…I insisted and insisted…and then, the truth is revealed [in a dramatic way]—‘Oh, my God you got to go to the hospital right away!’* (PR20). *I would've appreciated [the oncologist] pulling me out of the room and talking to me one‐on‐one…letting us know, ‘Hey, this is what's going on. Would you like me to talk to [child's name] or would you like to talk to her?’ And then I can come in and if she has any questions, let her ask those questions*…*in front of your child you don't have a chance, because you're just finding out, you don't have a chance to comfort them as they find out* (PR12). *Sometimes though I do wish somebody [was] watching him so that we could have conversations because it's hard to, if he's acting up and being fussy, it's hard to have those conversations* (PR21).
Imbalance between truth and hope	*[I was at] the county hospital for chemo. And whenever it stopped working, they just called my wife and told her that I had six months to live. Like we had a physical appointment that next day too. So it's not like you couldn't avoid it until an in‐person appointment, but now it's just—they just wanted to call and ruin our day and tell us that if I stop treatments that I'll die in six months. But if I want to continue with them, it's not going to be possible, because treatment isn't working. So it's like, okay. So you're telling me you're going to stop treating me, but also telling me if I stop treatment, I'm going to die* (PT1, age 24). *I don't like when people try to be like, “Well, at least you have something to look forward to with this”…or like, “Good news.” I know it was good news but it kind of made me [think], well, I have other tumors and stuff…I knew it was a positive thing to say but it didn't really make me feel great* (PT3, age 14). *[The oncologists] need to be cognizant of how they're telling you the facts. We get it that it's aggressive. We get it, that it may be hard to treat, but you still give hope* (PR20). *[The oncologist] goes, when this type of cancer comes back, the percentage of him surviving is 0 basically. And I go, are you really telling me that? And then she goes, well, it is based on studies and, you know, past years and how this cancer when it comes back, you know, story about it.” So for me, it was like, “So basically, you're telling me that he's gonna die even though I put him in treatment”* (PR22).

### 3.1 Insufficient Information

Patients and parents often described negative experiences with prognostic disclosure in the context of not receiving enough information about their disease status and what it meant for their future life. Within this theme, four concepts were identified as factors compounding patient/parent perceptions of insufficient information: (1) inadequate explanation or deficits in information provided; (2) vagueness or ambiguity of language; (3) deficits in anticipatory guidance; and (4) inconsistent availability of clinicians.

Patients (PT) and parents (PR) each described circumstances in which explanations about prognosis were inadequate or they perceived holes in the information provided. For example, patients discussed situations in which it was difficult to extract prognostic information. As one patient said:

It seemed like they just kept avoiding the questions I would ask…It was kind of hard to ask questions and get a proper answer…they also did not do a very good job of keeping us updated or telling us the entire story. It was a lot of fragmented information (PT1, age 24).

Both patients and parents also highlighted the use of vague or ambiguous language as contributing to their perception of poor‐quality prognostic disclosure. One patient wished that the oncologist would “*Be more straightforward and [come] out with everything*” (PT3, age 14). Another patient described inability to connect the dots when the oncologist did not provide specific information, saying, “*I feel like I don't completely understand everything, they kind of give an overview, but I'd really like to know what exactly is going on*” (PT2, age 18). Parents similarly viewed prognostic communication negatively when the oncologist was ambiguous, expressing frustration when “*[our oncologist] wouldn't communicate it directly to us where it would leave us wondering…we were still left in the back field, not knowin’ exactly what was going on*” (PR1). Another parent illustrated the confusion that resulted for their family as a result of vague prognostic disclosure:

You can tell it's a hard thing to say and you could tell [the oncologist] was having a hard time saying it. At some point, we had to go back and say, ‘So, do we know this is at Stage 4?’…We didn't blame her for that. It's hard to say, but it was confusing at first. We had to come out and ask, ‘Is this what you're saying?’ you know? (PR2).

Deficits in anticipatory guidance were also identified by both patients and parents as a challenge to effective prognostic disclosure. Parents, more than patients, had a negative perception of conversations in which the oncologist placed the burden of asking questions on the parent, without offering guidance on what questions to ask. One parent recalled, “Looking back in the past, I would've asked a question about that four months ago if I would've even known to ask a question about that” (PR3). The same parent also cautioned oncologists against saying “Do you have questions?” explaining that she “didn't even know what my questions were at the time.” One patient shared frustration about the lack of communication about what to expect in the long term: “There hasn't been a lot of in the future what we'll do. It's mostly just been what we're doing right now” (PT4, age 17). Another parent identified a similar tension between wanting to know more information about the future, even in the context of a likely incurable disease: “It's been kind of a day by day…It's hard to answer because they're not talking to us about how this will affect her college years…”. (PR4).

Additionally, parents more than patients emphasized inconsistent or insufficient access to clinicians as another driver of inadequate prognostic information. One parent summarized their disappointment in the dearth of information at critical timepoints in the child's illness, saying:

It's kind of hard to know when you're going to see your doctor, which has been kind of frustrating…sometimes you see your doctor and sometimes you just see a nurse…there's no real consistency to when you're getting information or when you can ask (PR5).

### 3.2 Overwhelming or Contradictory Information

Patients and parents also felt that receiving too much or conflicting information adversely impacted the quality of prognostic communication. Within this theme, three main drivers of negative prognostic disclosure emerged: (1) complex medical terminology; (2) excessive or out‐of‐place content; and (3) disjointed communication between multiple clinicians.

Both patients and parents underlined the use of jargon as a misstep that negatively influenced their perception of prognostic communication. As one patient described, most of the communication that did not go well centered around “*Medical words you don't really just understand unless you're a doctor*” (PT5, age 20). Parents reinforced this point, explaining that fear of being buried in complex medical terminology deters families from eliciting or engaging in prognostic communication: “It's like they're coming at it from too much of the doctor perspective…we don't want to ask and get a whole scientific answer” (PR5).

Parents more than patients specifically emphasized the harm incurred when oncologists share too much information at once or at an inopportune time. One parent described a negative reaction to information overload, saying, “*They told me a lot of things…And I don't think I was even able to digest all of that, because there's a whole lot…my mind was on my daughter. I was just praying for her to get better*” (P6). Another parent felt that certain types of prognostic disclosure, such as likelihood of infertility, should not be shared as an afterthought at the close of a visit:

We were very emotional already…[the oncologist] just came back into the room, she goes, ‘Oh, by the way, [child's name] won't be able to have babies after all this’…You really should just be concerned about your child's life, but then when all of a sudden you're hit with a reality of another thing that's going to affect her when and if she does live, it was just a lot (PR7).

Parents more than patients also stressed the damage caused by disjoined or conflicting communication from the medical teams. One parent shared their distress around the disclosure of contradictory information about prognosis: “[Some team members said] ‘Oh, her prognosis is good,’ and some other team members have been like, ‘Well, she's not responding the way we want’” (PR8).

### 3.3 Absence of Person‐Centered Connection

Primarily, parents described negative experiences with prognostic disclosure in which they felt dehumanized or felt that their individual needs and preferences were not honored. Three key contributors were identified exclusively by parents within this theme, driving their perceptions of negative prognostic disclosure: (1) lack of empathy or impersonal disclosure; (2) failure to center the parent's voice and/or preferences; (3) imbalance between truth and hope.

Parents described negative experiences with hearing prognostic information when they felt that the oncologist lacked empathy or was just “checking off a box,” rather than seeing her as a suffering human. One parent shared their pain when the oncologist said “there's nothing to do” without offering empathy: “They, off‐the‐rip, told me there's no cure, and they're not a research center, so they can't do it” (PR9). Another parent felt disenchanted by the oncologist's use of impersonal language, as if reading from a script, in a way that made her feel like someone in a play instead of a real person with feelings: “You know, that particular experience felt canned to me and like rehearsed at the same time” (PR10).

Parents also reported negative experiences related to the oncologist's failure to center the parent's voice and/or preferences. One parent shared feelings of frustration linked to their perception of being dismissed, expressing their wish that the oncologist “just sit down and listen to me” (PR11) Parents also disliked it when the oncologist shared information about prognosis in front of a child without giving the parent a chance to determine where/when/how to disclose prognosis to the child. Lack of consideration for parent preferences for how the patient engages with prognostic communication negatively impacted participants, who felt that this approach caused trauma or fear (“I look over at my daughter and she is terrified”) (PR12) and/or stole a parent's opportunity to mitigate distress (“For the first time, I would've appreciated them pulling me out of the room and talking to me one‐on‐one…in front of your child, you don't have a chance…to comfort them as they find out”) (PR12).

Finally, parents expressed how lack of balance between hope and realism can negatively impact prognostic communication. One parent underlined the importance of providing honest information while still creating space for hope, and described how their oncologist's approach did not offer any room to explore or reframe hopes: “*[The oncologist] was like, why are we even doing this*” (PR10). However, parents also recognized the importance of not making false promises, with one parent describing their oncologist's unrealistic optimism as harmful: “I feel like I was getting false hope. I want them to just come out and say it, so I can know, and I won't have to guess” (PR13).

## 4 Discussion

**FIGURE 1:**
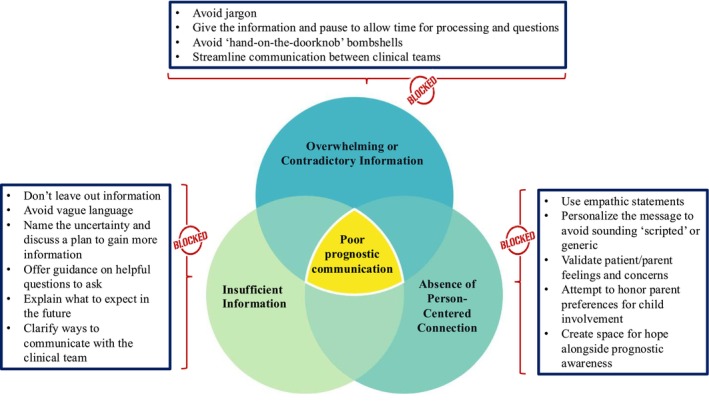
Clinical strategies to avoid prognostic disclosure “pitfalls” described by patients/parents. When patients/parents shared their negative experiences with prognostic disclosure, three main themes emerged, comprising 12 approaches (or gaps) that patients/parents perceived to be unhelpful in the setting of prognostic communication. From these findings, the authors extrapolated tangible strategies for prognostic disclosure to avoid common pitfalls identified by patients/parents who face poor prognosis illness.

Despite a growing emphasis on communication skills training in pediatric oncology training [20], prognostic disclosure remains a challenge for cancer care professionals [11]. This study revealed a relatively high incidence of poor prognostic communication perceived by parents of children with cancer, most of whom reported poor quality communication about what cancer means for the child's future life. Fewer patients reported negative experiences, although about one‐quarter did recall poor prognostic disclosure. Understanding patient/parent perceptions of what drives suboptimal prognostic disclosure offers unique learning opportunities for educators, trainees, and cancer care professionals to avoid ineffective communication approaches while developing and practicing alternative skills for high‐quality prognostic communication (Figure 1).

Insufficient information and overwhelming or contradictory information were both common drivers of poor‐quality prognostic disclosure for patients/parents, affirming existing literature that underscores patient and parent desire for comprehensive information exchange [21, 23, 24] or the need to “know everything the oncologist knows.” [26] This finding illuminates a common tension for oncologists who may struggle to balance sharing not enough versus too much information. Notably, oncologists infrequently ask patients and parents about their preferences for hearing information about prognosis [26], and this approach may help guide clinicians in personalizing the amount of information they share with a patient/parent at a given time.

With advances in cellular and targeted therapies, uncertainty about prognosis further complicates provision of clear prognostic disclosure. The path of least resistance may be to hedge, thereby avoiding incorrect predictions [10, 11]. Pediatric patients and parents identify “managing uncertainty” as a fundamental facet of quality communication, and these findings corroborate the critical importance of mastering this skill as ambiguous statements may cause distress for patients/parents. We encourage clinicians to acknowledge uncertainty with candor and vulnerability, while also describing the specific circumstances or next steps that are known. Clinicians might also consider asking patients/parents if they might prefer to hear a range of possibilities or best/worst‐case scenarios [31] to align prognostic disclosure with individualized preferences.

Although avoiding jargon is well described as a core communication skill in pediatric oncology [32], these findings suggest opportunities for improvement and the need for ongoing reinforcement of this skill within communication training. Moreover, while existing communication skills training programs often teach clinicians to ask “what questions do you have?”, these data suggest that this type of broad, unguided solicitation may exacerbate, rather than mitigate, anxieties for some patients/parents. Providing structured guidance on key questions may help alleviate anxiety and improve communication outcomes. Standardized question prompts or coaching phrases have been shown to be effective and may help support oncologists by offering language to open the door to a difficult conversation and lay a foundation on which to build a personalized message [33]. Finally, the data show that timing matters, and sharing critical information at inopportune moments, like the end of a visit, can overwhelm families. We encourage clinicians to assess in advance when, how, and what a given patient/parent wants to hear, create ample time and space for information processing, and respect family preferences for how information is shared with the child.

These data also affirm prior literature underscoring hope as highly meaningful to parents of children with cancer across the illness experience [34, 35, 36]. Oncologists often worry about striking the right balance between hope and frank disclosure [37], sometimes delaying or avoiding prognostic communication because they fear it will steal hope [38, 39]. However, hope and prognostic awareness can coexist [34], and open communication about poor prognosis does not lessen hope [40]. We advocate for oncologists to share information honestly while also creating room for hope, leveraging best practice recommendations on navigating the “breadth of hopes” after disclosing devastating news, whether by sharing in a family's hope for a miracle or asking “what else are you hoping for?” to reframe and support new hopes in the face of poor prognosis [41].

Notably, participating patients in this study were aged 12–24 years, representing a spectrum of timepoints across the lifespan. How different developmental ages and stages influence patients' perception of negative prognostic communication remains understudied, although data pertaining to broader communication preferences suggest that older adolescents and young adults often value their agency and may have different perceptions of good versus bad prognostic disclosure compared to pediatric or adult populations [21, 24]. Future research is needed to better understand prognostic communication preferences across the lifespan to help inform the development of clinical tools that encourage individualized prognostic disclosure.

This study has several limitations. Participants were recruited from a pediatric cancer center that specializes in early‐phase trials for relapsed/refractory disease, which may influence participant perspectives in unknown ways; however, participating patients and parents were likely to have experienced disease progression with multiple opportunities for prognostic disclosure, offering an in‐depth exploration of prognostic communication experiences to enrich the analysis. Fewer patients were enrolled compared to parents due to the inclusion of the bereaved parent cohort, and further research is needed to explore younger patient perspectives. Despite purposeful sampling to recruit a diverse population, most parents were mothers and more than half of the participants self‐identified as white; notably, this approach yielded greater numbers of fathers compared to prior research studies at the institution as well as the inclusion of Black and Hispanic participants at rates above institutional demographics. Finally, future work will prioritize the enrollment of participants who speak languages other than English to elevate diverse perspectives.

In summary, this study characterizes patient/parent perspectives on prognostic communication experiences and identifies opportunities to improve prognostic disclosure practices. Study findings yielded possible communication pitfalls to target during formal communication skills training as well as during experiential learning at the bedside, recognizing the need for individualized application of approaches within a given family or context. Future research will focus on collaboration with patients, parents, and multidisciplinary clinicians to codesign a clinical intervention to support the provision of person‐centered prognostic communication.

## Author Contributions

**Adriana Areizaga Ayala:** formal analysis (lead), writing – original draft (supporting), writing – review and editing (supporting). **Harmony Farner:** data curation (supporting), formal analysis (supporting), methodology (supporting), project administration (lead), supervision (supporting), writing – review and editing (supporting). **Shoshana Mehler:** formal analysis (supporting), methodology (supporting), writing – review and editing (supporting). **Caroline Christianson:** formal analysis (supporting), validation (supporting), writing – original draft (supporting), writing – review and editing (supporting). **Tara M. Brinkman:** conceptualization (supporting), funding acquisition (supporting), methodology (supporting), writing – review and editing (supporting). **Justin N. Baker:** conceptualization (supporting), funding acquisition (supporting), methodology (supporting), resources (supporting), writing – review and editing (supporting). **Pamela S. Hinds:** conceptualization (supporting), funding acquisition (supporting), methodology (supporting), writing – review and editing (supporting). **Jennifer W. Mack:** conceptualization (supporting), formal analysis (supporting), writing – review and editing (supporting). **Erica C. Kaye:** conceptualization (lead), data curation (lead), formal analysis (lead), funding acquisition (lead), investigation (lead), methodology (lead), project administration (lead), resources (lead), supervision (lead), validation (lead), visualization (lead), writing – original draft (lead), writing – review and editing (lead).

## Ethics Statement

This research protocol was approved by the Institutional Review Board at St. Jude Research Hospital.

## Conflicts of Interest

The authors declare no conflicts of interest.

## Precis

In the RIGHTime study, pediatric cancer patients and parents reported real‐world negative experiences with prognostic disclosure, and analysis revealed three main drivers of their perception of poor‐quality communication: Insufficient information; overwhelming or contradictory information; person‐centered connection. These data will inform future collaboration with patients, parents, and multidisciplinary cancer care professionals to codesign an intervention to support individualized prognostic disclosure in the context of advanced cancer.

## Supporting information


Data S1.

## Data Availability

In the context of the rarity of advancing pediatric cancer and the relatively small sample sizes intrinsic to qualitative research, a small risk exists for participant identification even following rigorous de‐identification procedures. Given this risk, our research team does not share entire raw data sets upfront to all‐comers. We are enthusiastic about sharing de‐identified data on a case‐by‐case basis to researchers under a data‐sharing agreement in the setting of an IRB approved research protocol and explicit assurance that data will be reviewed and analyzed exclusively for research purposes without identification of individual participants.
